# Chagas disease and SARS-CoV-2 coinfection does not lead to worse in-hospital outcomes

**DOI:** 10.1038/s41598-021-96825-3

**Published:** 2021-10-13

**Authors:** Israel Molina, Milena Soriano Marcolino, Magda Carvalho Pires, Lucas Emanuel Ferreira Ramos, Rafael Tavares Silva, Milton Henriques Guimarães-Júnior, Isaias José Ramos de Oliveira, Rafael Lima Rodrigues de Carvalho, Aline Gabrielle Sousa Nunes, Ana Lara Rodrigues Monteiro de Barros, Ana Luiza Bahia Alves Scotton, Angélica Aparecida Coelho Madureira, Bárbara Lopes Farace, Cíntia Alcantara de Carvalho, Fernanda d’Athayde Rodrigues, Fernando Anschau, Fernando Antonio Botoni, Guilherme Fagundes Nascimento, Helena Duani, Henrique Cerqueira Guimarães, Joice Coutinho de Alvarenga, Leila Beltrami Moreira, Liege Barella Zandoná, Luana Fonseca de Almeida, Luana Martins Oliveira, Luciane Kopittke, Luís César de Castro, Luisa Elem Almeida Santos, Máderson Alvares de Souza Cabral, Maria Angélica Pires Ferreira, Natália da Cunha Severino Sampaio, Neimy Ramos de Oliveira, Pedro Ledic Assaf, Sofia Jarjour Tavares Starling Lopes, Tatiani Oliveira Fereguetti, Veridiana Baldon dos Santos, Victor Eliel Bastos de Carvalho, Yuri Carlotto Ramires, Antonio Luiz Pinho Ribeiro, Freddy Antonio Brito Moscoso, Rogério Moura, Carísi Anne Polanczyk, Maria do Carmo Pereira Nunes

**Affiliations:** 1 PROSICS Barcelona, Vall d’Hebron University Hospital, Passeig de la Vall d’Hebron, 119, 08035 Barcelona, Spain; 2 Instituto René Rachou-FIOCRUZ Minas, Av. Augusto de Lima, 1715, Belo Horizonte, Brazil; 3 Department of Internal Medicine, Medical School and Telehealth Center, University Hospital, Universidade Federal de Minas Gerais, Avenida Professor Alfredo Balena 190 Sala 246, Belo Horizonte, Brazil; 4 Institute for Health Technology Assessment (IATS/ CNPq), Rua Ramiro Barcelos, 2359, Prédio 21 | Sala 507, Porto Alegre, Brazil; 5 Department of Statistics, Universidade Federal de Minas Gerais, Av. Presidente Antônio Carlos, 6627, ICEx, Sala 4071, Belo Horizonte, Brazil; 6 Hospital Marcio Cunha, Av. Tsunawaki Avenue, 41, Ipatinga, Brazil; 7 Medical School, Universidade Federal de Minas Gerais, Avenida Professor Alfredo Balena, 190, Belo Horizonte, Brazil; 8 Hospital UNIMED BH, Av. Do Contorno, Belo Horizonte, 3097 Brazil; 9 Hospital Metropolitano Doutor Célio de Castro, Rua Dona Luiza, 311, Belo Horizonte, Brazil; 10 Hospital Regional Antônio Dias, R. Maj. Gote, 1231, Patos de Minas, Brazil; 11 Hospital Risoleta Tolentino Neves, Rua das Gabirobas, 01, Belo Horizonte, Brazil; 12 Hospital João XXIII, Av. Professor Alfredo Balena, 400, Belo Horizonte, Brazil; 13 Hospital de Clínicas de Porto Alegre, Av. Ramiro Barcellos, 2350, Porto Alegre, Brazil; 14 Hospital Nossa Senhora da Conceição and Hospital Cristo Redentor, Av. Francisco Trein, 326, Porto Alegre, Brazil; 15 Hospital Julia Kubitschek, R. Dr. Cristiano Rezende, 2745, Belo Horizonte, Brazil; 16 Internal Medicine Department, University Hospital, Universidade Federal de Minas Gerais, Av. Prof Alfredo Balena, 110, Belo Horizonte, Brazil; 17 Universidade do Vale do Taquari (UNIVATES), Av. Avelino Talini, 171, Lajeado, Brazil; 18 Hospital Bruno Born, Av. Benjamin Constant, 881, Lajeado, Brazil; 19 Center for Research and Graduate Studies in Business Administration, Universidade Federal de Minas Gerais, Av. Presidente Antônio Carlos, 6627, Belo Horizonte, Brazil; 20 Grupo Hospitalar Conceição, Hospital Nossa Senhora da Conceição, Av. Francisco Trein, 326, Porto Alegre, Brazil; 21 Centro Universitário de Patos de Minas, R. Maj. Gote, 808, Patos de Minas, Brazil; 22 Hospital Eduardo de Menezes, R. Dr. Cristiano Rezende, 2213, Belo Horizonte, Brazil; 23 Pontífica Universidade Católica de Minas Gerais, Av. Dom José Gaspar, 500, Belo Horizonte, Brazil; 24 Medical School of Fundação Educacional do Município de Assis, Av. Getúlio Vargas, 1200, Assis, Brazil; 25 Hospital Balbino - Rede D`or São Luiz, R. Angélica Mota, 90, Rio de Janeiro, Brazil; 26 Internal Medicine Department, Universidade Federal do Rio Grande do Sul, Rua Ramiro Barcelos, 2359, Prédio 21 | Sala 507, Porto Alegre, Brazil; 27 Faculdade de Ciências Médicas de Minas Gerais, Alameda Ezequiel Dias, 275, Belo Horizonte, MG CEP 30130-100 Brazil

**Keywords:** Virology, SARS-CoV-2, Epidemiology, Microbiology, Cardiology, Diseases, Health care, Medical research

## Abstract

Chagas disease (CD) continues to be a major public health burden in Latina America. Information on the interplay between COVID-19 and CD is lacking. Our aim was to assess clinical characteristics and in-hospital outcomes of patients with CD and COVID-19, and to compare it to non-CD patients. Consecutive patients with confirmed COVID-19 were included from March to September 2020. Genetic matching for sex, age, hypertension, diabetes mellitus and hospital was performed in a 4:1 ratio. Of the 7018 patients who had confirmed COVID-19, 31 patients with CD and 124 matched controls were included (median age 72 (64–80) years-old, 44.5% were male). At baseline, heart failure (25.8% vs. 9.7%) and atrial fibrillation (29.0% vs. 5.6%) were more frequent in CD patients than in the controls (p < 0.05). C-reactive protein levels were lower in CD patients compared with the controls (55.5 [35.7, 85.0] vs. 94.3 [50.7, 167.5] mg/dL). In-hospital management, outcomes and complications were similar between the groups. In this large Brazilian COVID-19 Registry, CD patients had a higher prevalence of atrial fibrillation and chronic heart failure compared with non-CD controls, with no differences in-hospital outcomes. The lower C-reactive protein levels in CD patients require further investigation.

## Introduction

Since the first case of coronavirus disease 19 (COVID-19) described in Brazil on February 26th, 2020, SARS-CoV 2 infection has evolved as a global pandemic. The disease has a wide spectrum of clinical manifestations, ranging from asymptomatic cases to severe pneumonia and acute respiratory distress syndrome^1, 2^.

Although the great majority of symptoms are unspecified, mild, flu-like or belonging to respiratory sphere, other organs could be affected, as the cardiovascular system. COVID-19 has been associated with multiple cardiac manifestations, including cardiac arrhythmias, myocardial infarction, acute heart failure and acute fulminant myocarditis. Cardiovascular involvement has shown to be associated with increased mortality^3, 4^.

Underlying comorbidities have been widely associated with a worse prognosis for COVID-19 patients, since viral infections could act as triggers for worsening of chronic diseases^5–7^. Chagas disease (CD) is a multisystemic disorder, potentially affecting, cardiovascular, digestive, and neurological systems. It is the most common cause of infectious cardiomyopathy worldwide, and it may play a role in the clinical prognosis of COVID-19 patients^8, 9^. Although CD is endemic in Latin America, it has been recognized that the disease is now a worldwide concern, as the disease spread with population movements from endemic to non-endemic countries^10^. In Brazil, CD still remains a public health challenge, being one the countries with more absolute number of patients and an annual incidence rate of approximately 0.16 per 100,000 inhabitants/year^11^.

Potential interactions between COVID-19 and Chagas cardiomyopathy could be probable, because both conditions share the same immunological pathway. SARS-CoV-2 spike proteins bind to angiotensin-converting enzyme-2 (ACE-2), which is needed to invade the host cell. On the other hand, ACE2 is involved in heart function and the development of hypertension and diabetes mellitus (DM), risk factors frequently observed in patients with chronic Chagas cardiomyopathy^12, 13^. Those patients could have increased levels of ACE2 because of the chronic use of ACE inhibitors and/or angiotensin receptor blockers (ARBs).

Limited information is available regarding the characteristics and outcomes of patients with CD and COVID-19. Therefore, we aim to describe the characteristics, laboratory, and imaging findings, as well as in-hospital outcomes of CD and COVID-19 coinfected patients included in the Brazilian COVID-19 Registry.

## Methods

This manuscript adheres to the Strengthening the Reporting of Observational Studies in Epidemiology (STROBE) guideline^14^. All methods were performed in accordance with the relevant guidelines and regulations.

### Study design and subjects

Patients were selected from the Brazilian COVID-19 Registry, a prospective multicenter cohort project with 37 participant hospitals in 17 cities from three Brazilian states (Minas Gerais, Pernambuco, Rio Grande do Sul, Santa Catarina, São Paulo). Details of the cohort were published elsewhere^5^.

COVID-19 diagnosis was confirmed through real time polymerase-chain reaction (RT-PCR) nasopharyngeal and oropharyngeal swab testing or anti-SARS-CoV-2 IgM detected in serological assay in serum or plasma sample, according to World Health Organization guidance^15^.

For the present study, patients with previous history of CD recorded in the database were selected. CD diagnosis were retrieved by their own hospital record or self-referred by the patient. Patients were admitted from March 1 to September 30, 2020. At the moment of the analysis 7018 patients were introduced in the registry, 31 of those were classified as suffering from CD.

### Data collection

Study data were collected by trained hospital staff or interns using Research Electronic Data Capture (REDCap) tools^16^. Medical records were reviewed to collect data on patients’ demographic and clinical characteristics, including age, sex, pre-existing medical conditions and home medications; COVID-19 symptoms at hospital presentation; clinical assessment upon hospital admission, third and fifth admission days; laboratory, imaging, electrocardiographic data; inpatient medications, treatment and outcomes. Definitions were published elsewhere^5^.

### Patient and public involvement

This was an urgent public health research study in response to a Public Health Emergency of International Concern. Patients or the public were not involved in the design, conduct, interpretation or presentation of results of this research.

### Statistical analysis

Genetic matching for sex, age, hypertension, DM and hospital was performed in a 4:1 ratio (MatchIt package in R). Genetic matching is a multivariate matching method that uses an evolutionary search algorithm to determine the weight each covariate is given, to maximize the balance of observed covariates across individuals of both groups^17^. Sample size of 132 controls was calculated considering and expected risk ratio for mortality 2.5 in CD-group, power of 80%, alfa-error probability of 5% for a 4:1 CD/control.

Categorical data were presented as absolute numbers and proportions, and continuous variables were expressed as medians and interquartile ranges. The χ^2^ and Fisher Exact test were used to compare the distribution of categorical variables, and the Wilcoxon-Mann–Whitney test for continuous variables. Results were considered statistically significant if the two-tailed p-value was < 0.05. All statistical analysis was performed with R software (version 4.0.2).

### Ethics

The study was approved by the National Commission for Research Ethics (CAAE 30350820.5.1001.0008). Individual informed consent was waived by the National Commission for Research Ethics owing to the pandemic situation and the use of deidentified data, based on medical chart review only.

### Transparency declaration

The lead authors (MSM, IM and MCP) affirm that the manuscript is an honest, accurate, and transparent account of the study being reported; that no important aspects of the study have been omitted; and that any discrepancies from the study as originally planned (and, if relevant, registered) have been explained.

## Results

### Patient characteristics at hospital admission

**Figure 1:**
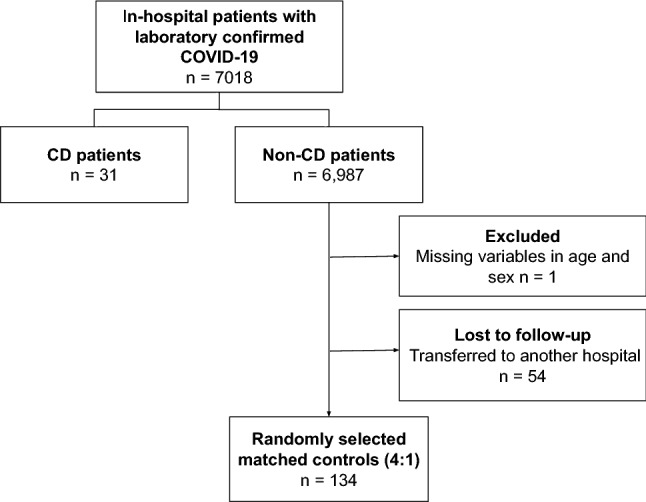
Flowchart of COVID-19 patients included in the study.

From the 155 patients included in the study (Fig. 1), 31 were reported as having Chagas disease, and 124 were matched controls. The median age was 72.0 (64.0–79.5) years-old and 44.5% were male. Hypertension (65.8%), DM (32.3%), chronic obstructive pulmonary disease (COPD) in (16.7%), chronic heart failure (12.9%) and atrial fibrillation (10.3%) were the most frequent comorbidities. All patients were diagnosed for COVID-19 through a positive RT-PCR for SARS-CoV-2.

Patients were from 11 hospitals, with average 382 beds (ranging from 60 to 936 beds). Nine of them (81.8%) were public, 7 (63.6%) were teaching hospitals and 8 (72.7%) were reference centers for COVID-19 treatment.

When comparing CD patients with controls (Table 1), there were no significant differences in demographic and medical characteristics, except for the prevalence of chronic heart failure (8 [25.8%] vs 12 [9.7%]; p = 0.031) and atrial fibrillation (9 [29.0%] vs 7 [5.6%]; p < 0.001), which were more prevalent in CD patients. Although the median number of comorbidities was higher in CD patients (3.0 [2.0, 4.0] vs. 2.0 [1.0, 3.0]), this difference did not reach statistical significance (p = 0.119).

**Table 1:** Demographic characteristics and medical history data of the study population at baseline.

	CD patients (n = 31)	Controls (n = 124)	p-value
Age* (years)	74.0 (64.5, 79.0)	72.0 (64.0, 80.0)	0.856
Male sex*	14 (45.2%)	55 (44.4%)	> 0.999
Comorbidities**			
Total number			0.461
0	3 (9.7%)	11 (8.9%)	
1	3 (9.7%)	27 (21.8%)	
2	9 (29.0%)	39 (31.5%)	
3	7 (22.6%)	26 (21.0%)	
4	6 (19.4%)	16 (12.9%)	
≥ 5	3 (9.7%)	5 (4.0%)	
Cardiovascular diseases			
Hypertension*	20 (64.5%)	82 (66.1%)	> 0.999
Ischemic cardiopathy	1 (3.2%)	6 (4.8%)	> 0.999
Chronic heart failure	8 (25.8%)	12 (9.7%)	0.031
Atrial fibrillation/flutter	9 (29.0%)	7 (5.6%)	< 0.001
Stroke	2 (6.5%)	8 (6.5%)	> 0.999
Pacemaker	1 (3.2%)	0 (0.0%)	0.200
Respiratory diseases			
Asthma	1 (3.2%)	9 (7.3%)	0.688
COPD	8 (25.8%)	18 (14.5%)	0.216
Metabolic diseases			
Diabetes mellitus*	10 (32.3%)	40 (32.3%)	> 0.999
Obesity (BMI > 30)	1 (3.2%)	10 (8.1%)	0.695
Other conditions			
Cirrhosis	0 (0.0%)	2 (1.6%)	> 0.999
Psychiatric condition	1 (3.2%)	9 (7.3%)	0.688
Chronic renal disease	0 (0.0%)	3 (2.4%)	> 0.999
Dyslipidemia	0 (0.0%)	1 (0.8%)	> 0.999
HIV	0 (0.0%)	2 (1.6%)	> 0.999
Neoplasia	3 (9.7%)	8 (6.5%)	0.461
Transplantation	1 (3.2%)	3 (2.4%)	> 0.999
Dementia	0 (0.0%)	1 (0.8%)	> 0.999
Epilepsy	0 (0.0%)	0 (0.0%)	–
**Toxic habits**			
Alcohol	1 (3.2%)	6 (4.8%)	> 0.999
Tobacco (active or former)	7 (22.6%)	35 (28.2%)	0.684

Numbers are presented are medians (P25-P75) or counts (percentages).

*BMI* body mass index, *CD* Chagas disease, *COPD* chronic obstructive pulmonary disease.

*Controls were paired for age, sex, hospital, hypertension and diabetes.

**This variable does not include Chagas disease.

The median time since from symptom onset to hospital admission was 6 (8–4) days. Dyspnea and cough (dry or productive) were present in more than one half of patients. There were no differences in the clinical presentation between both groups (Table 2).

**Table 2:** Clinical characteristics of the study population at baseline.

	CD patients (n = 31)		Controls (n = 124)		p-value
	Frequency (%) or median (IQR)	Valid cases	Frequency (%) or median (IQR)	Valid cases	
**Symptoms**					
Time from symptom onset	5.0 (3.0, 7.8)	30	6.0 (3.8, 9.2)	124	0.392
Adynamic	10 (32.3%)	31	37 (29.8%)	124	0.965
Ageusia	4 (12.9%)	31	7 (5.6%)	124	0.232
Anosmia	5 (16.1%)	31	10 (8.1%)	124	0.183
Headache	7 (22.6%)	31	22 (17.7%)	124	0.719
Rhinorrhea	4 (12.9%)	31	20 (16.1%)	124	0.786
Diarrhea	3 (9.7%)	31	18 (14.5%)	124	0.573
Dyspnea	19 (61.3%)	31	73 (58.9%)	124	0.967
Odynophagia	14 (45.2%)	31	64 (51.6%)	124	0.659
Fever	4 (12.9%)	31	17 (13.7%)	124	> 0.999
Hyporexia	1 (3.2%)	31	5 (4.0%)	124	> 0.999
Neurological manifestations	6 (19.4%)	31	34 (27.4%)	124	0.491
Myalgia	2 (6.5%)	31	19 (15.3%)	124	0.252
Nausea/vomiting	7 (22.6%)	31	21 (16.9%)	124	0.639
Productive cough	18 (58.1%)	31	65 (52.4%)	124	0.717
Dry cough	1 (3.2%)	31	1 (0.8%)	124	0.361
**Clinical assessment**					
Glasgow < 15	6 (19.4%)	31	24 (19.4%)	124	> 0.999
HR	80.0 (72.0, 86.8)	30	84.0 (77.0, 96.0)	121	0.060
HR ≥ 100 bpm	4 (12.9%)	31	28 (22.6%)	124	0.346
RR	22.0 (18.5, 26.0)	27	22.0 (18.0, 25.0)	115	0.748
RR ≥ 24 irpm	16 (51.6%)	31	56 (45.2%)	124	0.658
Sat O2	94.0 (91.0, 96.0)	29	94.0 (90.0, 96.0)	123	0.712
Sat O2 < 90%	7 (22.6%)	31	28 (22.6%)	124	> 0.999
SF ratio	402.4 (300.0, 440.5)	28	395.8 (240.0, 438.1)	123	0.316
Invasive ventilation	3 (9.7%)	31	13 (10.5%)	124	> 0.999
SBP ≤ 100 mmHg	1 (3.2%)	31	11 (8.9%)	124	0.462
Inotropic drugs	12 (38.7%)	31	45 (36.3%)	124	0.967

*CD* Chagas disease, *HR* hear rate, *IQR* interquartile range, *RR* respiratory rate, *SF ratio* Sat O_2_/FiO_2_, *valid cases* non missing cases.

Laboratory and imaging findings are presented in Supplementary Table S1 and S2. Median C-reactive protein was lower in CD patients than the controls (55.5 [35.7, 85.0] vs. 94.3 [50.7, 167.5] mg/dL). There was no other clinically relevant difference in laboratory exams between groups.

At admission, diffuse interstitial infiltrate pattern and ground glass opacities were the most prevalent findings in the chest X-ray and chest computer tomography (CT), respectively. No significant differences were found in the frequency of abnormalities and radiological progression in both groups, expect for the frequency of pleural effusion in the follow-up CT, more frequent in CD patients.

Among CD, patients 10 had an EKG performed. Of those, 4 patients had atrial fibrillation and 2 had a pacemaker rhythm, so the proportion of patients with sinus rhythm in controls were significantly higher than in CD patients (68.8% vs 40.0%, p = 0.142) (Table 3).

**Table 3:** Electrocardiographic characteristics of the study population at baseline and new abnormalities at follow-up.

	CD patients (n = 31)	Control patients (n = 124)	p-value
**ECG at admission**	10 (32.3%)	32 (26.0%)	0.637
Sinus rhythm	4 (40.0%)	22 (68.8%)	0.142
Atrial fibrillation or flutter	4 (40.0%)	7 (21.9%)	0.410
Pacemaker	2 (20.0%)	1 (3.1%)	0.136
Right bundle branch block	1 (10.0%)	4 (12.5%)	> 0.999
Left bundle branch block	2 (20.0%)	1 (3.1%)	0.136
Left ventricular hemiblock	0 (0.0%)	0 (0.0%)	
New electrocardiographic abnormalities*	N* = 4 (12.9%)	N* = 15 (12.3%)	> 0.999
Rhythm			
Atrial fibrillation or flutter	4 (100.0%)	6 (40.0%)	0.087
Pacemaker	1 (25.0%)	0 (0.0%)	0.211
Multifocal atrial rhythm	0 (0.0%)	1 (6.7%)	> 0.999
Supraventricular tachycardia	0 (0.0%)	1 (6.7%)	> 0.999
Monomorphic ventricular tachycardia	0 (0.0%)	3 (20.0%)	> 0.999
Polymorphic ventricular tachycardia	0 (0.0%)	1 (6.7%)	> 0.999
No new rhythm abnormalities	0 (0.0%)	4 (26.7%)	0.530
New long QTc interval	1 (25.0%)	2 (13.3%)	0.530
None	2 (50.0%)	4 (26.7%)	0.557

*New electrocardiographic abnormalities through in-hospital follow-up, and number of patients in which this outcome was assessed. *CD* Chagas disease, *ECG* electrocardiogram, *QTc* corrected QT interval.

### Treatment and clinical outcomes

There were no differences regarding the therapeutic strategy among both groups (Table 4), except for a trend of higher frequency of therapeutic anticoagulation in CD patients (19.3% vs. 10.5%, p = 0.206). Twenty-four CD patients (77.4%) and 103 controls (83.0%) received corticosteroids (p = 0.448). Dexamethasone was used by 64.5% CD patients and 66.1% controls (p > 0.999). Macrolides were prescribed for 77.4% in CD patients and 87.1% controls (p = 0.255); chloroquine or hydroxychloroquine in 3.2% and 4.8% (p > 0.999). Only one patient received remdesivir.

**Table 4:** Medications.

	CD patients (n = 31)	Controls (n = 124)	p-value
Azithromycin	23 (74.2%)	91 (73.4%)	> 0.999
Clarithromycin	1 (3.2%)	17 (13.7%)	0.126
Chloroquine	0 (0.0%)	1 (0.8%)	> 0.999
Hydroxycloroquine	1 (3.2%)	5 (4.0%)	> 0.999
Remdesivir	0 (0.0%)	2 (1.6%)	> 0.999
**Anticoauglation**			
Profilatic			
Low-molecular-weight	16 (51.6%)	65 (52.4%)	> 0.999
Non-fractioneted	11 (35.5%)	58 (46.8%)	0.353
Fondaparinoux	0 (0.0%)	1 (0.8%)	> 0.999
Therapeutic	0 (0.0%)	1 (0.8%)	> 0.999
Low-molecular-weight	5 (16.1%)	8 (6.5%)	0.138
Non-fractioneted	1 (3.2%)	5 (4.0%)	> 0.999

During hospitalization, 72 (46.5%) of patients required admission to the intensive care unit, and among them 55 (35.4%) needed mechanical ventilation and 26 (16.8%) substitutive renal therapy. Overall, there were no differences in in terms of clinical evolution and outcomes (Table 5).

**Table 5:** Clinical outcomes.

	CD patients (n = 31)		Control patients (n = 124)		p-value
	Frequency (%) or median (IQR)	Valid cases	Frequency (%) or median (IQR)	Valid cases	
Length of stay	8.0 (4.5, 13.5)	31	10.0 (7.0, 17.0)	124	0.220
**Admission to ICU**	16 (51.6%)	31	56 (45.2%)	124	0.658
Time from admission to ICU (days)	1.0 (0.0, 2.0)	16	0.5 (0.0, 2.0)	56	0.891
Days in ICU	6.0 (2.0, 11.2)	16	7.5 (4.0, 14.0)	56	0.352
Thromboembolic events	0 (0.0%)	31	7 (5.6%)	124	0.346
Mechanical ventilation	10 (32.3%)	31	45 (36.3%)	124	0.834
Acute kidney injury	9 (37.5%)	24	45 (41.7%)	108	0.884
RRT	5 (16.1%)	31	21 (16.9%)	124	> 0.999
Sepsis	6 (19.4%)	31	24 (19.4%)	124	> 0.999
Nosocomial infection	3 (9.7%)	31	24 (19.4%)	124	0.314
Acute heart failure	2 (6.5%)	31	5 (4.0%)	124	0.628
Acute respiratory distress	9 (29.0%)	31	44 (35.5%)	124	0.641
Death	10 (32.3%)	31	38 (30.6%)	124	> 0.999

*CD* chagas disease, *ICU* intensive care unit, *IQR* interquartile range, *RRT* renal replacement therapy.

## Discussion

We described a cohort of CD patients infected with SARS-COV-2 and admitted in hospitals belonging to a large Brazilian COVID-19 Registry project. Overall, CD patients had similar clinical characteristics and outcomes to non-CD controls, matched by age, sex, hypertension, DM and hospital, except from a higher prevalence of atrial fibrillation and chronic heart failure, and lower C-reactive protein levels.

Due to the potential cardiac involvement, and the higher procoagulant state, *T.cruzi* and SARS-COV-2 coinfection has been postulated as condition for myocardial damage, depression of ventricular function, increased arrhythmogenic state, thromboembolism risk, and ultimately a worst prognosis^18–20^. However, it was only a hypothesis and no previous study has tested it using patient data. Despite the limited number of patients with CD (31) our study refuted did not confirm the hypothesis. We did not find any significant difference or even a trend of worse clinical outcomes in CD patients, even with a higher frequency of atrial fibrillation and heart failure in the CD group.

Current data demonstrates that SARS-CoV-2 infection induces immune dysfunction, widespread endothelial injury, complement-associated coagulopathy and systemic microangiopathy^21^. By the other hand, *T. cruz*i infection is associated with an upregulated procoagulant activity in plasma. Therefore, it could be expected a greater risk of thromboembolic manifestations. In our cohort the overall thrombosis event was 4.5% (7 out of 155), all of them were in the control group. Noteworthy that, the great majority of patients (91%) were treated with oral anticoagulants because its underlying disease or received any kind of prophylactic heparin when admitted to the hospital, as recommended by national and international guidelines for the management of in-hospital COVID-19 patients^22, 23^.

The lower median C-reactive level in CD patients was an unexpected finding. We hypothesize that CD patients, as they already have an active chronic inflammatory and immune response triggered by *T. cruzi* infection, might have a lower risk of unregulated inflammatory response to COVID-19^24^. Therefore, what could have been a factor for worse prognosis, due to a higher frequency of associated heart failure and atrial fibrillation and the CD itself, could be equilibrated by a controlled inflammatory response. This is only a hypothesis, that merits consideration for future studies. If proved correct, it may add to the knowledge of understating how to prevent the unregulated inflammatory response in COVID-19.

It is also interesting to discuss the influence that the use of anticoagulants in full doses may have had on the outcomes of patients with CD and COVID-19. The higher prevalence of atrial fibrillation in those patients may had led to a higher frequency of use of therapeutic dosage anticoagulants (19.3% vs. 10.5%), which did not reach statistical significance due to the sample size. The best strategy to be used—prophylactic or therapeutic heparin doses—in patients with moderate to severe COVID-19 is not yet defined, and it has been hypothesized that therapeutic anticoagulation (full dose heparin) is associated with decreased in-hospital mortality in patients with moderate COVID-19, but not in patients with severe COVID-19.

It is known the effect of immunosuppressant drugs and the risk of reactivation of CD. In the case of corticosteroids, immunosuppressive doses have not been associated with higher rates of reactivation of CD, although is controversial due to the lack of supporting evidence^25, 26^. Tocilizumab, a cytokine inhibitor (recombinant humanized monoclonal antibody with an antagonist effect on the IL-6 receptor), combined with another immunosuppressant agents have been suggested to be associated with the reactivation of latent infections, including parasites.

Two published case reports of Strongyloides Hyperinfection Syndrome in COVID-19 patients immunosuppressed with dexamethasone and tocilizumab, have been recently published^27, 28^. To date, no cases of CD reactivation have been published, but at least, there is a concern that COVID-19 disease therapeutics could potentially trigger reactivation of CD. This merits further investigation and until definitive evidence is published, it should be a cause of concern in decision making, when prescribing immunosuppressors in these patients﻿.

The fact that the majority of CD patients were admitted to public hospitals (81.8%) is an indicator that CD disproportionally affects people from lower income background. In a previous multivariate analysis, we demonstrated that despite being admitted to public hospitals patients do not have worse prognosis than patients admitted to private ones^5^.

This study has limitations. In addition to the retrospective design, subject to the drawbacks of a patient records review, the number of CD was low. However, it is the largest series published to date. Due to the pragmatic study design, laboratory and imaging tests were performed at the discretion of the treating physician. In that sense, Chagas disease diagnosis was based on medical records or by self-reporting, in these cases no extra serology was performed. Despite the limited representativity of radiologic, tomographic and electrocardiographic analysis, no patient performed echocardiogram during hospital admission.

## Conclusions

Although coinfection by *Trypanosoma cruzi* and SARS-COV-2 may pose a risk of complications and therefore a worse prognosis, in our series we did not find significant differences in terms of clinical presentation and outcomes of patients with CD compared to controls, despite a higher frequency of chronic heart failure and atrial fibrillation at baseline. We observed lower C-reactive protein levels in CD when compared to controls, and this merits further investigation.

## Supplementary Information

Supplementary Information.

## Data Availability

Data are available upon reasonable request.
