# Supplementary material for: Chagas disease and SARS-CoV-2 coinfection does not lead to worse in-hospital outcomes
Source: Sci Rep. 2021 Oct 13;11:20289. doi: 10.1038/s41598-021-96825-3 (PMC8514447; doi:10.1038/s41598-021-96825-3)
Supplement: Supplementary file 1 — Supplementary Information. [file 41598_2021_96825_MOESM1_ESM.pdf]

Table S1. Laboratory parameters of the study population at baseline

	CD patients (n=31)		Controls (n=124)		p-value
	Frequency (%) or median (IQR)	Valid cases	Frequency (%) or median (IQR)	Valid cases	
Hemoglobin (g/dL)	12.8 (12.1, 14.1)	28	12.9 (11.8, 14.0)	120	0.849
White blood cell count (x10^9/L)	6.3 (4.7, 8.0)	28	7.0 (5.1, 10.0)	120	0.205
Neutrophils (x10^9/L)	4,142.5 (3,287.5, 6,102.2)	28	5,548.5 (3,528.8, 7,597.5)	118	0.154
Lymphocytes (x10^9/L)	960.0 (609.8, 1,443.8)	28	1,001.0 (688.2, 1,291.5)	116	0.824
Platelets (x10^9/L)	221.5 (147.3, 319.8)	28	194.2 (138.3, 272.5)	119	0.186
Albumin (g/dL)	3.3 (2.9, 3.7)	13	3.2 (2.9, 3.6)	50	0.792
AST (U/L)	45.0 (28.8, 50.5)	20	46.0 (33.0, 69.0)	79	0.351
ALT (U/L)	26.5 (14.0, 38.2)	20	30.0 (21.8, 47.0)	80	0.154
Calcium (mmol/L)	1.1 (1.0, 1.2)	10	1.1 (1.1, 1.1)	39	0.302
Creatinine (mg/dL)	1.0 (0.9, 1.2)	27	0.9 (0.7, 1.3)	117	0.546
CPK (U/L)	87.5 (64.0, 183.0)	10	107.0 (47.0, 306.0)	41	0.553
C-reative protein (mg/dL)	55.5 (35.7, 85.0)	28	94.3 (50.7, 167.5)	115	**0.013**
D-dimer (ng/mL)	1.9 (1.1, 600.0)	17	4.0 (1.9, 1,765.0)	68	0.106
LDH (U/L)	435.0 (311.0, 471.5)	19	381.5 (285.0, 515.2)	76	0.930
Lactate (mmol/L)		31		123	0.863
Potassium (mmol/L)	4.3 (3.8, 4.7)	27	4.3 (3.9, 4.7)	116	0.782
aPTT (seconds)	1.1 (1.0, 1.4)	22	1.0 (0.9, 1.1)	87	**0.004**
INR	1.2 (1.1, 1.6)	23	1.1 (1.0, 1.2)	89	**0.018**
Sodium (mmol/L)	137.0 (133.9, 139.8)	26	137.0 (133.6, 141.0)	115	0.856
Total bilirubin (mg/dL)	0.4 (0.3, 0.6)	18	0.5 (0.3, 0.7)	83	0.866
Troponin (ng/mL)	0.6 (0.5, 3.5)	9	0.6 (0.1, 1.0)	40	0.366

Urea (mg/dL)	40.5 (32.8, 59.8)	28	42.0 (31.0, 67.2)	118	0.673

Table S2. Radiological characteristics of the study population at baseline

	CD patients (n=31)	Controls (n=124)	p-value
**Chest X-ray at admission**	**19 (61.3%)**	**91 (73.4%)**	0.269
Normal	3 (15.8%)	19 (20.9%)	0.760
Atelectasis	0 (0.0%)	2 (2.2%)	>0.999
Consolidation	6 (31.6%)	15 (16.5%)	0.195
Diffuse interstitial infiltrate	9 (47.4%)	52 (57.1%)	0.599
Focal interstitial infiltrate	1 (5.3%)	6 (6.6%)	>0.999
Bilateral ground glass opacity	6 (31.6%)	17 (18.7%)	0.223
**Chest X-ray at follow up**	**11 (35.5%)**	**58 (46.8%)**	0.353
Normal	3 (27.3%)	9 (15.5%)	0.390
Atelectasis	0 (0.0%)	2 (3.4%)	>0.999
Consolidation	2 (18.2%)	17 (29.3%)	0.715
Diffuse interstitial infiltrate	5 (45.5%)	33 (56.9%)	0.525
Focal interstitial infiltrate	0 (0.0%)	4 (6.9%)	>0.999
Bilateral ground glass opacity	6 (54.5%)	14 (24.1%)	0.067
Radiological progression	3 (27.3%)	11 (19.0%)	0.683
**Chest CT at admission**	**8 (25.8%)**	**48 (38.7%)**	0.259
Normal	1 (12.5%)	3 (6.2%)	0.470
Atelectasis	2 (25.0%)	3 (6.2%)	0.144
Consolidation	2 (25.0%)	9 (18.8%)	0.649
Pleural effusion	0 (0.0%)	2 (4.2%)	>0.999
Bilateral ground glass opacity	6 (75.0%)	40 (83.3%)	0.623
Peripheral ground glass opacity	5 (62.5%)	25 (52.1%)	0.712
Crazy-paving pattern	0 (0.0%)	6 (12.5%)	0.578
**Chest CT at follow up**	**5 (16.1%)**	**31 (25.0%)**	0.419
Normal	0 (0.0%)	5 (16.1%)	>0.999
Atelectasis	1 (20.0%)	2 (6.5%)	0.370
Consolidation	0 (0.0%)	7 (22.6%)	0.559

Pleural effusion	3 (60.0%)	2 (6.5%)	**0.013**
Unilateral ground glass opacity	0 (0.0%)	2 (6.5%)	>0.999
Bilateral ground glass opacity	3 (60.0%)	19 (61.3%)	>0.999
Peripheral ground glass opacity	3 (60.0%)	19 (61.3%)	>0.999
Crazy-paving pattern	2 (40.0%)	6 (19.4%)	0.305
Progression*	5 (100.0%)	30 (96.8%)	>0.999

* Normal at admission and abnormal at follow-up

CT: computer tomography.
